# Two-step phosphorylation of Ana2 by Plk4 is required for the sequential loading of Ana2 and Sas6 to initiate procentriole formation

**DOI:** 10.1098/rsob.170247

**Published:** 2017-12-20

**Authors:** Nikola S. Dzhindzhev, George Tzolovsky, Zoltan Lipinszki, Mohammed Abdelaziz, Janus Debski, Michal Dadlez, David M. Glover

**Affiliations:** 1 Department of Genetics, University of Cambridge, Cambridge CB2 3EH, UK; 2 MTA SZBK Lendület Laboratory of Cell Cycle Regulation and Institute of Biochemistry, Biological Research Centre, Hungarian Academy of sciences, Szeged, Hungary; 3 Laboratory of Mass Spectrometry, Institute of Biochemistry and Biophysics, Polish Academy of Sciences, 02-106 Warsaw, Poland

**Keywords:** Ana2, Plk4, Sas6, centriole, centrosome

## Abstract

The conserved process of centriole duplication requires Plk4 kinase to recruit and promote interactions between Sas6 and Sas5/Ana2/STIL (respective nomenclature of worms/flies/humans). Plk4-mediated phosphorylation of Ana2/STIL in its conserved STAN motif has been shown to promote its interaction with Sas6. However, STAN motif phosphorylation is not required for recruitment of Ana2 to the centriole. Here we show that in *Drosophila*, Ana2 loads onto the site of procentriole formation ahead of Sas6 in a process that also requires Plk4. However, whereas Plk4 is first recruited to multiple sites around the ring of zone II at the periphery of the centriole, Ana2 is recruited to a single site in telophase before Plk4 becomes finally restricted to this same single site. When we over-ride the auto-destruction of Plk4, it remains localized to multiple sites in the outer ring of the centriole and, if catalytically active, recruits Ana2 to these sites. Thus, it is the active form of Plk4 that promotes Ana2's recruitment to the centriole. We now show that Plk4 phosphorylates Ana2 at a site other than the STAN motif, which lies in a conserved region we term the ANST (ANa2-STil) motif. Mutation of this site, S38, to a non-phosphorylatable residue prevents the procentriole loading of Ana2 and blocks centriole duplication. Thus the initiation of procentriole formation requires Plk4 to first phosphorylate a single serine residue in the ANST motif to promote Ana2's recruitment and, secondly, to phosphorylate four residues in the STAN motif enabling Ana2 to recruit Sas6. We discuss these findings in light of the multiple Plk4 phosphorylation sites on Ana2.

## 1. Introduction

Centrioles are the core components of centrosomes and their regulated duplication is critical to ensure cells have a single centrosome at each of their spindle poles during the cell division cycle [1–3]. Centrioles also become the basal bodies of cilia, which are required for many aspects of cell signalling and motility [1,4]. Thus, the dysregulation of centriole function or duplication is associated with a wide range of inherited diseases and with oncogenic transformation [1,5–7]. Plk4 is a master regulator of centriole duplication; loss of Plk4 leads to loss of centrioles [8,9] (and its overexpression can lead to their de novo formation and over-duplication [10–12]). Plk4 is targeted to the centrosome through its interaction with either or both Spd2/Cep192 and Asl/Cep152 depending upon the species [13–17]. The kinase is known to auto-phosphorylate a degron sequence to direct its SCF-mediated self-destruction [18–23]. Failure of this process leads to excessive Plk4 and centriole over-duplication.

Centriole duplication is initiated through an interaction between two conserved proteins first identified in *Caenorhabditis elegans*, Sas5 and Sas6 [13,14]. Dimers of Sas6's counterparts are known in other species to assemble into ninefold symmetrical structures that form the structural basis of the cartwheel that is assembled upon the initiation of procentriole formation [24,25]. Sas6 is recruited to the procentriole in *Drosophila* and human cells in a process that requires Plk4 to phosphorylate several residues in the conserved STAN motif in the C-terminal part of the respective counterparts of Sas5, *Drosophila* Ana2 and human STIL [26–28]. In *Drosophila*, this acts as a binary switch to control Sas6 recruitment to the procentriole as soon as mother and daughter centrioles disengage in late telophase [26]. Mutation of four phosphorylation sites in the STAN motif of *Drosophila* Ana2 into alanine residues prevents recruitment of Sas6 to the site of procentriole formation while still permitting the centriolar recruitment of this mutant variant of Ana2. The process governing the recruitment of Ana2 independently of Sas6 is thus unclear. In human cells, it has been shown that continued activity of Plk4 is required for the recruitment of STIL to the centriole but the molecular basis for this is not understood [28]. Here, we show that the recruitment of Ana2 to the nascent procentriole also requires the presence and activity of Plk4 and occurs in response to the phosphorylation of Ana2 at a conserved serine residue in its N-terminal domain. Our results indicate a two-step mechanism whereby phosphorylation of Ana2 in its N-terminal part promotes its recruitment to the site of procentriole formation and its phosphorylation in the STAN motif leads to the subsequent recruitment of Sas6.

## 2. Material and methods

### 2.1. DNA constructs

All cDNA and expression constructs and cloning methods were previously described [15,26].

### 2.2. Cell culture, DNA and dsRNA transfections

D.Mel-2 cells (originally from Thermo Fisher Scientific) were cultured and treated with dsRNA as described previously [15]. Transfections of DNA constructs were performed as described previously [26]. Stable cell lines were established as reported [29]. Primers for generating dsRNA were all reported elsewhere [15,18,26,30], except for the following:

Sas4-F: 5′-GAATTAATACGACTCACTATAGGGAGAATGCAGGAGGCTGGCGAAAGTCC -3′
Sas4-R: 5′-GAATTAATACGACTCACTATAGGGAGAGGAGGCTTCATCATCGGCATGAG -3′

### 2.3. Site-directed mutagenesis

Generation of *Ana2* point-mutations was either as already reported [26], or by using the QuikChange II XL Site-Directed Mutagenesis Kit (Agilent) on cDNA or entry clones as template and the oligonucleotide primers given in the electronic supplementary material, table S1.

### 2.4. Recombinant protein expression and purification

All recombinant proteins in this study together with the methodology for their expression and purification from *Escherichia coli* have been described elsewhere [26].

### 2.5. *In vitro* Plk4 phosphorylations

*In vitro* phosphorylation of ^35^S-methionine-labelled Ana2 produced by coupled *in vitro* transcription–translation (IVTT) and of GST-Ana2 proteins on beads were carried out as previously described [26].

### 2.6. Lambda phosphatase treatment

D.Mel-2 cells were co-transfected with pAct5-Ana2-FLAG and either pAct5-Plk4-NDKD (kinase-dead) or pAct5-Plk4-ND (active) in a 12-well plate. Approximately 1 × 10^6^ cells were collected from each well 24 h post-transfection and briefly rinsed in PBS. Cells were then lysed in RIPA buffer (50 mM Tris–HCl pH 7.4; 150 mM NaCl; 1% NP-40; 0.5% Na deoxycholate; 0.1% SDS; 1× Complete EDTA-free protease inhibitor cocktail tablets from Roche) on ice for 15 min. Lysates were cleared by centrifugation and supplemented with 1× Lambda-phosphatase buffer and 1× MnCl_2_ solution provided along with Lambda phosphatase (New England Biolabs, catalogue number P0753S). Two samples (one of Ana2-FLAG + Plk4-NDKD and one of Ana2-FLAG + Plk4-ND) were mock-treated (no phosphatase added), while one sample (Ana2-FLAG + Plk4-ND) was treated with 200 U (0.5 µl) Lambda phosphatase for 30 min at 30°C. All samples were then boiled in Laemmli sample buffer and analysed by immunoblotting.

### 2.7. Mass-spectrometry and phospho-peptide mapping

Phospho-peptide identification by mass spectrometry was carried out as previously explained [26]. Briefly, Ana2 (tagged with GST and pre-phosphorylated by Plk4 *in vitro*, or tagged with GFP-, FLAG- or Protein-A and purified from either D.Mel-2 cells or 0–3 h syncytial stage embryos) was digested with trypsin directly on the affinity resin. Twenty per cent (v/v) of the resulting peptide mixture was directly analysed by LC/MS and the remaining 80% enriched for phosphatides using titanium dioxide. Samples were analysed using an Orbitrap-LTQ mass spectrometer (Thermo Fisher Scientific) coupled to a UPLC system (Waters Corporation). Acquired data were searched using the Mascot Search Engine (Matrix Science) against the *Drosophila melanogaster* database. Phosphorylated peptides identified by Mascot were individually verified by manually inspecting the relevant spectra.

### 2.8. Antibodies

The following primary antibodies were used for immuno-fluorescence (IF) or western blotting (WB): rabbit-anti-Ana2 [26] (IF 1 : 1000); rat-anti-Sas6 [26] (IF 1 : 1000); chicken-anti-D-Plp [10] (IF 1 : 1000); mouse-anti-FLAG (clone M2, Sigma, WB 1 : 20 000); mouse-anti-Myc (clone 9E10, Abcam, WB 1 : 5000). Affinity-purified rabbit-anti-Plk4 (IF 1 : 100) was a kind gift from Dr Monica Bettencourt-Dias (Instituto Gulbenkian de Ciencia, Oeiras, Portugal).

### 2.9. Immunostaining and structured illumination microscopy

This was carried out as previously described [15,26]. In brief, D.Mel-2 cells were grown for 2–4 h on concanavalin A-coated coverslips and then fixed in cold methanol. Fixed cells were blocked in PBS, supplemented with 0.1% Triton X-100 and 10% fetal calf serum, incubated first with primary antibodies, washed in PBS, and then incubated with secondary antibodies. After several final washes in PBS supplemented with 0.1% Triton X-100, specimens were mounted in Vectashield containing DAPI (Vector laboratories). Super-resolution microscopy and image-analysis was performed on an OMX-V3 system using a 63×/1.4NA oil Olympus lens. Images (512 × 512 ppi) were reconstructed and registered using the SoftWorx Linux package. Images were further processed to obtain maximum intensity projections. These were cropped and assembled in Photoshop v6.

Stages of the cell cycle were assessed from the staining of chromosomes. We assigned stages to telophase from the positions of the centrosomes in relation to the long axis of the dividing cells immobilized on concanavalin A. We observed that in early telophase, centrosomes are positioned on the distal side of the nucleus to the spindle microtubules. As telophase progresses, the centrosomes appear to begin to migrate (mid-telophase) relative to the spindle such that they move towards the spindle-proximal side of the nucleus at the time of the first indication of cytokinetic furrow formation (late telophase). Owing to the adhesion of cells onto the concanavalin A-coated coverslips, the abscission stage of cytokinesis cannot be completed. Thus cells having a long cytokinetic bridge can also be in G1 phase. We classify such cells as in cytokinesis/G1.

To deplete centriole components from D.Mel-2 cells for structured illumination microscopy, we carried out RNAi for 5 days (except for 3 days in the case of Plk4 RNAi), as a result of which 30–60% of cells had no centrosomes. Our analysis was then carried out upon cells in which a single centrosome persists showing the defects we document.

## 3. Results

### 3.1. Ana2 loads ahead of Sas6 at the pre-procentriole

In our previous study, we followed the centriolar recruitment of both Sas6 and Ana2 relative to the conversion of daughter centrioles to centrosomes monitored by the extension of D-Plp around the periphery of the daughter centriole during progression through mitosis [26]. This revealed the ring of D-Plp around the daughter to be completed by late anaphase whereupon mother and daughter centrioles disengaged in anaphase/early telophase. We observed that following centriole disengagement both Sas6 and Ana2 were recruited to the site of procentriole formation. Our earlier study also showed that replacing endogenous Ana2 with a variant in which the four Plk4 phosphorylation sites in the STAN motif were replaced with alanine residues would prevent Sas6 from being recruited to the procentriole. Nevertheless this four-alanine STAN motif mutant could still itself be loaded indicating that phosphorylation of the STAN motif is not required for Ana2 loading.

**Figure 1.**
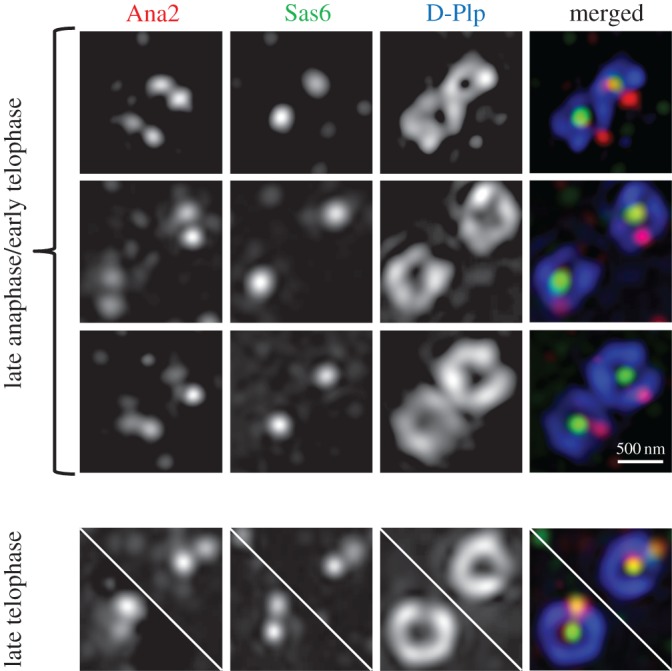
Ana2 loading onto centrioles occurs ahead of Sas6. Representative images of centrioles stained to reveal D-Plp (blue), Ana2 (red) and Sas6 (green) from D.Mel-2 cells in the indicated cell cycle stages. In late anaphase/early telophase, Ana2 is loaded onto both mother centrioles and at the sites of the nascent procentrioles, with Sas6 only on the mothers. In late telophase/cytokinesis, Ana2 and Sas6 are both on mother and at pre-procentriolar sites.

The above finding led us to ask whether in the normal course of centriole duplication, Ana2 could recruit to the procentriole ahead of Sas6 or whether, alternatively, Ana2 and Sas6 load together. To address this, we co-stained cells to reveal D-Plp, Sas6 and Ana2 and re-examined their relative loading focusing upon cells at the very late stages of anaphase/early telophase using structured-illumination (SIM), super-resolution microscopy. At the earliest stages of centriole disengagement, we were only able to observe single dots of Sas6 staining at the core of mother and daughter centrioles (Zone I [31]),while at this stage two dots of Ana2 staining were visible, one at the centriole core and the other at the site of the nascent procentriole (figure 1). From late telophase onwards, we could see two dots corresponding to Sas6 and Ana2 at both the core of the mother and daughter and at the site of the procentriole formation upon each of these disengaged centrioles. Thus, Ana2 is loaded onto the procentriole ahead of Sas6.

### 3.2. Loading of Ana2 onto the procentriole requires Asl and Plk4

**Figure 2.**
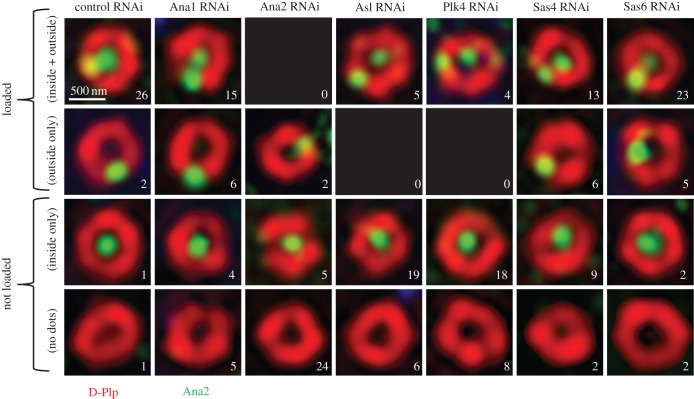
Ana2 recruitment to centrioles is dependent on Asl and Plk4. Representative structured illumination images of centrioles stained to reveal D-Plp (red) and Ana2 (green), following a 5-day depletion of the indicated protein by RNAi (3 days in the case of Plk4). Approximately 30 centrioles from cells in cytokinesis/G1 were examined for each condition and were scored in the categories shown as ‘loaded’ and ‘not loaded’. The number of centrioles in each category is indicated in each panel. The terms ‘loaded/not loaded’ are used here to reflect the effective presence/absence of Ana2 in the (D-Plp) ring of the centriole.

Our previous findings that the STAN-phospho-site mutant Ana2 can still load onto the procentriole site and that this occurs independently of Sas6 binding [26] led us to ask which centriolar proteins might be required for Ana2 loading. To address this question, we depleted cultured *Drosophila* cells of various centriole components by RNAi and used SIM to assess whether Ana2 was present at a single parental site encircled by D-Plp or also at the pre-procentriole site on the periphery of the D-Plp ring. We scored a minimum of 30 centrioles in cells that were in cytokinesis or very early G1, that is at a cell cycle stage at which procentrioles should have been established (figure 2). This revealed that 93% of control cells (28/30) had Ana2 recruited to the pre-procentriole site at this stage. A similar proportion (88%; 28/32) also had Ana2 at the site of the procentriole following RNAi treatment to deplete Sas6, in accord with our previous study [26]. The proportion of Ana2 at the procentriole site was somewhat reduced following depletion of Ana1 or Sas4 (70%; 21/30 and 63%; 19/30) although the greater proportion of centrioles still had Ana2 at the single peripheral, pre-procentriolar site. We cannot exclude the possibility that failure to recruit Ana2 in these circumstances is a secondary consequence of the known effects of these treatments upon centriole to centrosome conversion [30] or building the microtubule wall [14,32,33]. By contrast, depletion of either Plk4 or its loading factor, Asl, dramatically reduced loading of Ana2 to the procentriole to 13% (4/30) and 17% (5/30) of cells, respectively. Thus, we conclude that Plk4 is essential for the loading of Ana2 onto the site of procentriole formation and that this is independent of a second requirement for Plk4 to phosphorylate Ana2 in the STAN motif in order to bind and recruit Sas6.

### 3.3. Centriolar distribution of Plk4 does not define the single site of Ana2-loading

**Figure 3.**
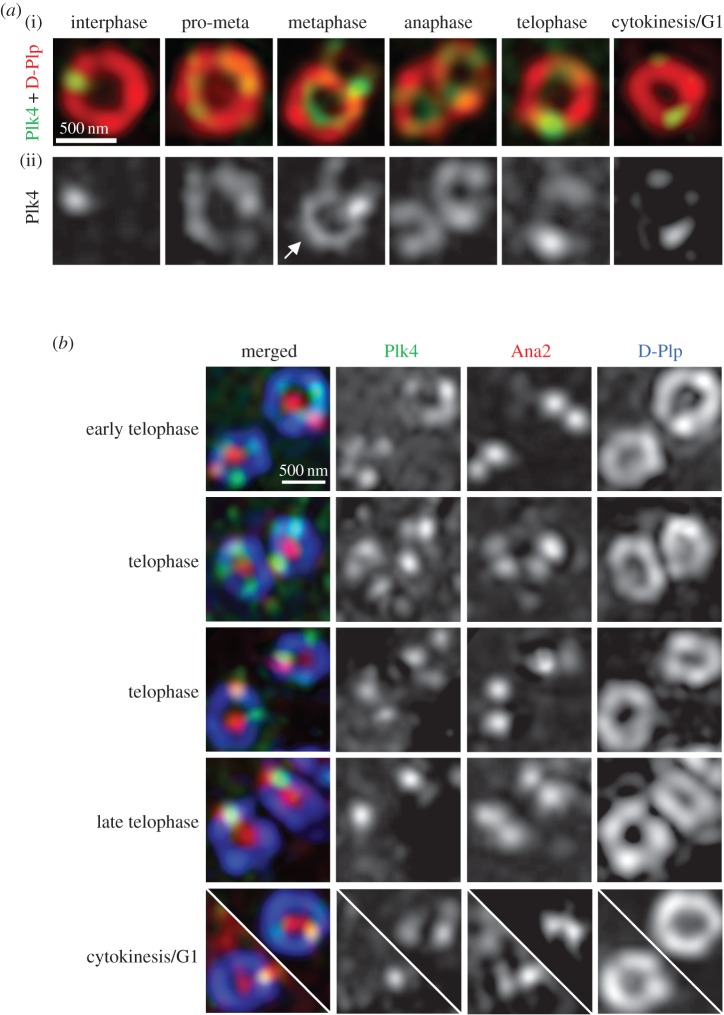
Centriolar distribution of Plk4 does not define the single site of Ana2-loading in telophase. (*a*) Distribution of Plk4 at centrioles throughout mitotic progression. Representative structured illumination images of centrioles stained to reveal D-Plp (red) and endogenous Plk4 (green, (i)) and monochrome (ii). In mitotic progression, the mother centriole (arrow) can be distinguished from the daughter prior to anaphase because of a more complete ring of staining given by antibodies against D-Plp/Plk4. (*b*) Centriolar distribution of Plk4 and Ana2 during telophase. Representative structured illumination images stained to reveal endogenous D-Plp (blue), Plk4 (green) and Ana2 (red). Note that at the stage of typical Ana2-loading (early telophase, top panel), Plk4 is still present in multiple dots in the form of a broken ring at the centriole periphery. The newly loaded Ana2 co-localizes with one of the peripheral Plk4 dots, which is not necessarily the Plk4 dot showing the strongest signal.

The requirement for Plk4 for the loading of Ana2 at the centriole led us to examine the relative positions of the two proteins on the centriole at the time of procentriole formation. It has been described that in mammalian cells, Plk4 accumulates in a ring around parental centrioles that resolves into a single dot, reportedly around the time when STIL and Sas6 are recruited for cartwheel formation [17,27]. We observed that in *Drosophila* cells, Plk4 also accumulated in progression through mitosis as a ring around the mother and daughter centrioles as the latter underwent conversion into a centrosome (figure 3*a*). During progression through telophase, the ring of Plk4 around the mother and daughter gradually broke down such that by cytokinesis/the following G1, it had resolved into a dot. To relate the distribution of Plk4 to recruitment of Ana2, we assessed the localization of the two proteins with respect to D-Plp in those centrioles in which Ana2 could be observed both at the centriole core/Zone I and at the nascent procentriole (figure 3*b*). We found that at the earlier stages of telophase, centrioles had Plk4 located in a variable number of beads arranged in a ring around the parental centriole, one of which coincided with newly loaded Ana2. Later, in cytokinesis/G1, centrioles had a single Plk4 bead colocalized with Ana2. Thus, Ana2 becomes localized to a single procentriole site ahead of the resolution of Plk4 from its ring-like to dot-like distribution.

### 3.4. Plk4 activity is critical for Ana2 loading

**Figure 4.**
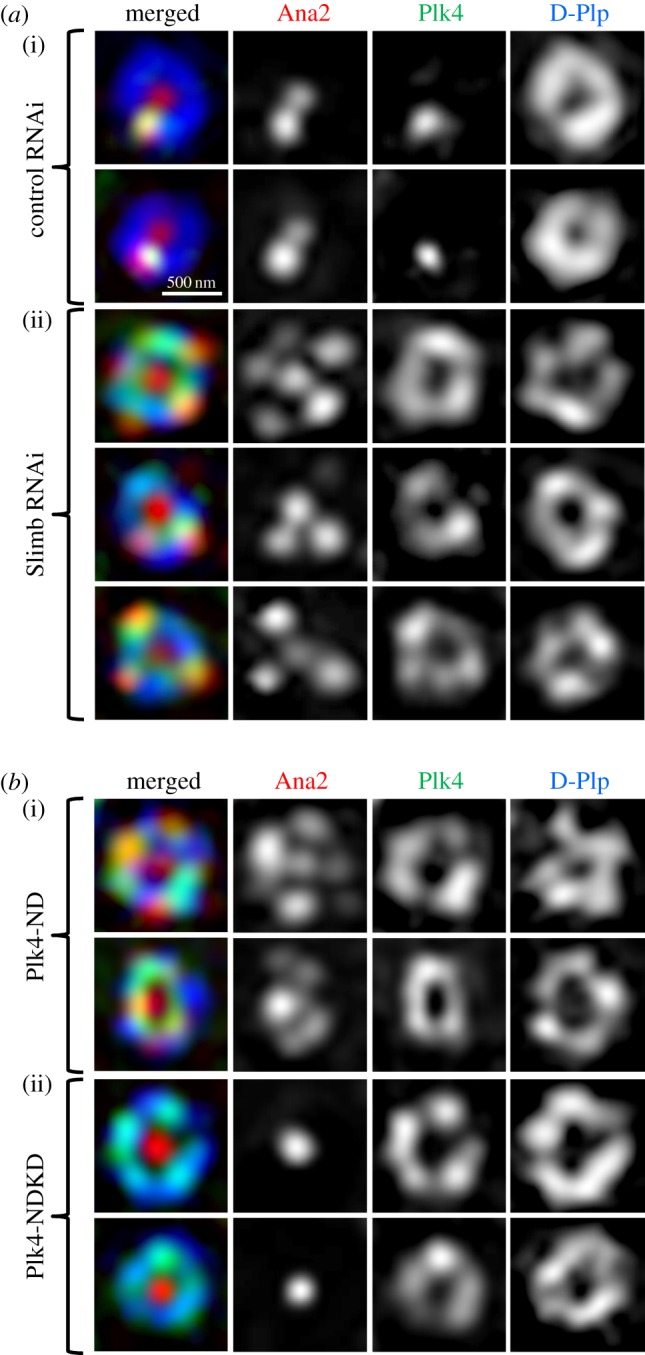
Plk4 activity is critical for Ana2-loading. (*a*) Depletion of the F-box protein, Slimb, stabilizes Plk4 at the centriole ring to recruit rosettes of Ana2 in interphase. Structured illumination images of interphase centrioles from cells subjected to 5 days of treatment with control dsRNA (against GST) or dsRNA directed against Slimb. Cells are stained to reveal endogenous D-Plp (blue), Plk4 (green) and Ana2 (red). (*b*) Overexpression of active, but not kinase-dead, Plk4 induces Ana2 loading at multiple sites. Structured illumination images of centrioles in interphase cells following 24 h of induced expression of either active non-degradable Plk4 (ND, (i)) or kinase-dead non-degradable Plk4 (NDKD, (ii)). Cells were stained to reveal endogenous D-Plp (blue), Ana2 (red) and overexpressed un-tagged Plk4 (green). Both active and inactive Plk4-ND accumulate in ectopic rings but only active Plk4 is able to induce rosettes of Ana2.

Normally, the levels of Plk4 are limited during interphase in both fly and mammalian cells by the SCF ubiquitin-protein-ligase that targets the proteasome-mediated destruction of the kinase by binding to its auto-phosphorylated degron [18–23]. This can be over-ridden in several ways: by over-expressing Plk4; by expressing a non-degradable (ND) Plk4 variant with a mutated degron; or by disrupting the SCF complex, which targets Plk4. This led us to ask whether Ana2 would be recruited to multiple sites if the levels of active Plk4 were increased. To this end, we first depleted the Plk4-targeting F-box protein of the SCF complex, which in *Drosophila* is encoded by *slimb* [18,34]. We found that, as a consequence, Plk4 accumulated in rings together with D-Plp in Zone III of interphase centrioles and that Ana2 was indeed recruited to multiple sites on these Plk4-positive rings (figure 4*a*). We then investigated the effects of over-expressing either kinase active or inactive mutant forms of Plk4-ND. We found that both forms of the ND kinase accumulated ectopically in rings in Zone III of the centrioles in interphase. However, only the active Plk4-ND was able to induce loading of Ana2 to multiple sites, while conversely the kinase dead Plk4-NDKD not only did not induce these ‘rosettes’ but even suppressed the normal loading of Ana2 as a single dot (figure 4*b*). Thus it seems that when Plk4 is continually present as a result of over-riding its auto-destruction, it can accumulate on the entire outer ring of the centriole and, if it is catalytically active, it can recruit Ana2 to multiple sites. Together, these results suggest that there is only one sufficiently active focus of Plk4 that exceeds a threshold for Ana2 loading.

### 3.5. Plk4 phosphorylates Ana2 at the conserved serine 38

Together, the above observations suggested that the Plk4-dependent recruitment of Ana2 to initiate procentriole formation might reflect an ability of Plk4 to phosphorylate Ana2. We have previously described four sites in the STAN motif of *Drosophila* Ana2 that when phosphorylated by Plk4 enable the binding and consequent centriole recruitment of Sas6. However, failure to phosphorylate Ana2 at these sites does not prevent its loading to centrioles [26]. As our above findings indicated that active Plk4 is required to recruit Ana2 to the centriole prior to Sas6 binding, we argued that other Plk4 sites within Ana2 must be responsible for this recruitment and wished to identify such sites.

**Figure 5.**
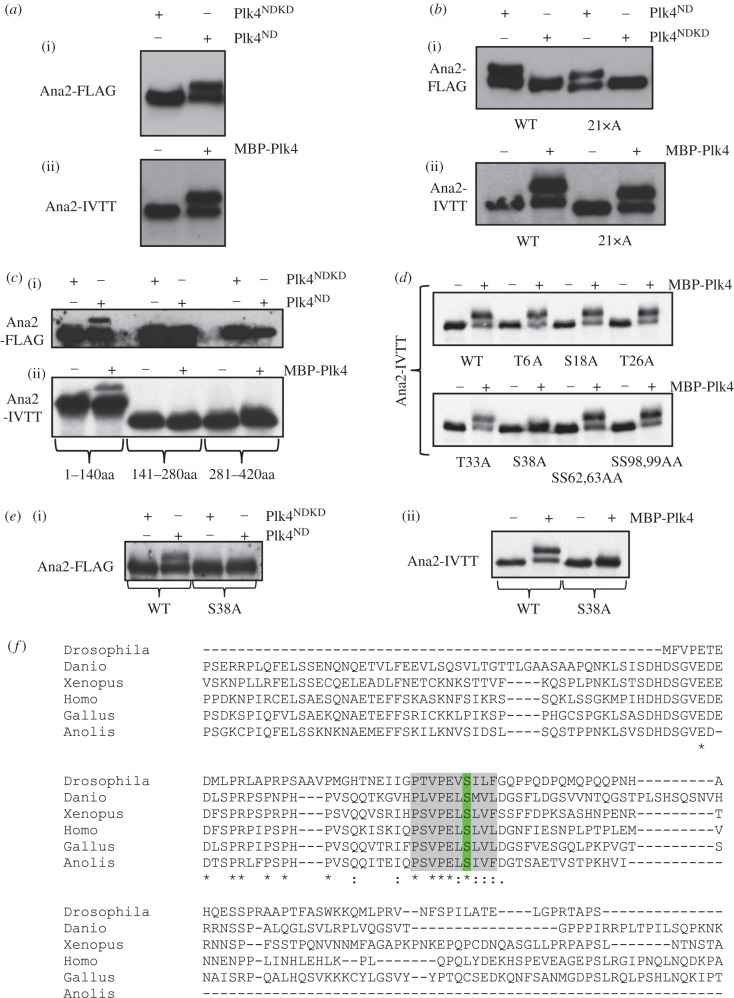
Plk4 phosphorylates Ana2 at conserved serine 38. (*a*) (i) Transient co-overexpression of Ana2-FLAG with active or kinase-dead non-degradable Plk4 (Plk4^ND^ or Plk4^NDKD^ respectively) in D.Mel-2 cells. Cell extracts were subjected to western blotting to reveal the Ana2-FLAG protein. (ii) ^35^S-Met-Ana2 protein synthesized by coupled transcription–translation *in vitro* and incubated with MBP-Plk4 and ATP *in vitro* and analysed by SDS-PAGE and autoradiography. Plk4-mediated phosphorylation *in vivo* or *in vitro* results in a shift in the electrophoretic mobility of Ana2. (*b*) Phosphorylation of Ana2 in which all 21 *in vitro* phosphorylation sites (highlighted in blue in electronic supplementary material, figure S1D) have been mutated to alanine residues. Assays are carried out using either the *in vivo* co-overexpression assay (i) or the *in vitro* phosphorylation assay (ii). None of the 21 mutations abolishes the band-shift. (*c*) The phosphorylation site responsible for the band shift is located within the N-terminal 140 amino acids of Ana2. Of the three Ana2 fragments, Ana2^1–140^, Ana2^141–280^ and Ana2^281–420^, only Ana2^1–140^ displays the band-shift in both the co-overexpression (i) and the *in vitro* phosphorylation assay (ii). (*d*) An S38A-Ana2 mutation, but not mutations in other candidate sites in the N-terminal 140 amino acids, abolishes the band-shift resulting from incubation with Plk4 *in vitro*. (*e*) The S38A mutation abolishes the shift in electrophoretic mobility of Ana2 following phosphorylation by Plk4 in either the *in vivo* co-overexpression assay (i) or in the *in vitro* assay (ii). (*f*) S38 (green) lies within a conserved region of Ana2—the ANST motif (highlighted grey). Partial alignment of *Drosophila melanogaster* Ana2 (top line) with STIL orthologues from *Danio rerio*, *Xenopus laevis*, *Homo sapiens*, *Gallus gallus* and *Anolis carolinensis*.

We had noticed that Ana2 undergoes a profound shift in its electrophoretic mobility following phosphorylation by Plk4 (figure 5*a*; electronic supplementary material, figure S1*a*). This mobility shift was seen when either FLAG-tagged-Ana2 was introduced into cultured *Drosophila* cells together with active Plk4 in transient transfections or when Ana2 synthesized *in vitro* by coupled transcription and translation (Ana2-IVTT) was phosphorylated by Plk4 *in vitro* (figure 5*a*). The mobility shift was reversed by Lambda phosphatase treatment indicating that it is a consequence of Ana2's phosphorylation (electronic supplementary material, figure S1B). Moreover, the mobility shift was still observed when the four Plk4-phospho-sites in the STAN motif were mutated to alanine residues indicating that it occurred independently of the mechanism required to recruit Sas6 (electronic supplementary material, figure S1C). We argued that such a dramatic mobility shift should reflect a significant conformational change in Ana2 and hypothesized that this might be required to recruit Ana2 to the centriole. We therefore sought to identify the phosphorylation site(s) responsible for the mobility shift.

The strategy we adopted to identify the site is primarily presented in the electronic supplementary material, figure S1. Briefly, we first subjected bacterially expressed GST-Ana2 to phosphorylation by recombinant Plk4 *in vitro* and identified 21 phosphorylated serine or threonine residues by mass spectrometry including the four STAN motif sites (residues highlighted in blue; electronic supplementary material, figure S1D). When we mutated all 21 sites to alanine residues, Ana2 continued to undergo a band shift following phosphorylation by Plk4 (figure 5*b*) indicating that none were responsible for this conformational change. We then used mass spectrometry to identify 12 phospho-sites in Protein-A- or FLAG-tagged Ana2, affinity purified from cultured cells, or in GFP-Ana2, affinity purified from *Drosophila* embryos (residues highlighted in yellow; electronic supplementary material, figure S1D). Having mapped the region undergoing the band shift to the N-terminal 280 residues (electronic supplementary material, figure S1E), we mutated the 12 sites to alanine residues and repeated the kinase assay, but none of the changes abolished the band shift (electronic supplementary material, figure S1F). We further narrowed down the region of Ana2 undergoing the band shift to within residues 1 and 140 (figure 5*c*) and then systematically mutated each individual remaining serine or threonine residue within this segment to alanine prior to phosphorylation of the corresponding IVTT peptide by Plk4 (figure 5*d*). This revealed that phosphorylation on serine 38 was responsible for the mobility shift (figure 5*d,e*). Suspecting that we had originally failed to detect this modification in our mass spectrometric analysis because it lay within a poorly ionized peptide, we then searched for low abundance ionized fragments within this region following phosphorylation of Ana2 by Plk4. The fragmentation pattern of a peptide extending from residue 13 to 65, which had a phospho-serine residue at position 38 (electronic supplementary material, figure S2), allowed us to confirm that Ana2 is phosphorylated by Plk4 on serine 38 to change its electrophoretic mobility. Strikingly, this residue lies within a 10-residue motif that is highly conserved from insects to vertebrates (figure 5*f*), which we refer to as the ANST motif.

Finally, we wished to determine whether phosphorylation of Ana2 on S38 was required for its subsequent interaction with Sas6 that we have previously shown to require Plk4-mediated phosphorylation of its STAN motif [26]. To this end, we produced GST-tagged forms of wild-type and S38A Ana2 in *E. coli* and immobilized the recombinant proteins on beads for treatment with active or kinase-dead Plk4. We then asked whether S^35^-labelled Sas6, generated by coupled IVTT, could then bind to the beads. This revealed that Sas6 was able to bind to both wild-type and S38A Ana2 but only after phosphorylation by Plk4 (electronic supplementary material, figure S3). Thus mutation of S38 in the ANST motif to an amino acid that cannot be phosphorylated does not affect the Plk4-dependent binding of Ana2 to Sas6.

### 3.6. Phosphorylation of Ana2 at S38 is required to load Ana2 for centriole duplication

**Figure 6.**
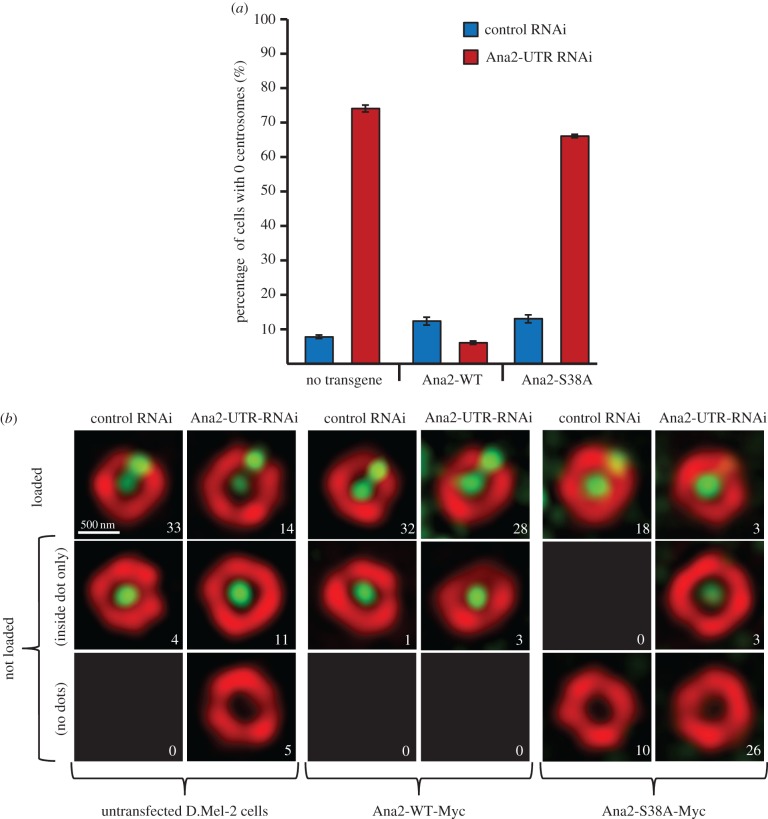
S38 is essential for centriole duplication and Ana2 loading. (*a*) Control D.Mel-2 cells and cells expressing either Ana2-WT or Ana2-S38A transgenes from the constitutive *Actin-5* promoter were treated with either control dsRNA or dsRNA targeting the UTRs of endogenous Ana2 (Ana2-UTR RNAi). Cells completely lacking centrosomes were scored after three rounds of treatment. Bars represent s.d. (*b*) Structured illumination images of centrioles stained with anti-D-Plp (red) and anti-Ana2 antibodies (in the case of untransfected D.Mel-2 cells, green) or anti-Myc (in the case of cells expressing Myc-tagged Ana2 transgenes) following three rounds of 4-day treatment with the indicated dsRNAs. Approximately 30 centrioles from interphase cells were scored for each condition and allocated to the categories ‘loaded’ and ‘not loaded’. The number of centrioles in each category is indicated in each panel.

To test our hypothesis that the phosphorylation event that induced the mobility shift of Ana2 would be important for the biological function of the protein, we mutated this residue to alanine (Ana2-S38A) and first tested the consequences for centriole duplication. To this end, we used three rounds of RNAi directed against the 3′ UTR of the Ana2 mRNA to deplete the endogenous Ana2 protein and block centriole duplication (figures 6*a*). We found that the loss of centrosomes resulting from this treatment could be rescued in cells expressing wild-type Ana2 lacking the endogenous UTR but not in cells expressing Ana2-S38A. Therefore, phosphorylation of Ana2 at S38 by Plk4 is required for centriole duplication.

We then asked whether S38 phosphorylation was required for the loading of Ana2 onto centrioles. Once again, we used three rounds of RNAi directed against the UTR of Ana2 to downregulate the endogenous protein and found that the proportion of centrioles in which Ana2 was loaded onto the procentriole site on the D-Plp ring was reduced from 89% (33/37) to 46% (14/30) (figure 6*b*). Stable expression of Myc-tagged wild-type Ana2 was able to rescue loading following this treatment to 90% (28/31). By contrast, Myc-tagged Ana2-S38A failed to rescue loading (9%; 3/32) of Ana2. Moreover, it also had a dominant-negative effect on loading: compare 64% (18/28) loading in control RNAi-treated cells expressing Ana2-S38A to 89% (33/37) loading in control-depleted untransfected cells indicating that Ana2-S38A is a stable protein as confirmed by western blotting (electronic supplementary material, figure S4). Thus, we conclude that phosphorylation of Ana2 at S38 is required for the loading of Ana2 to permit centriole duplication.

## 4. Discussion

**Figure 7.**
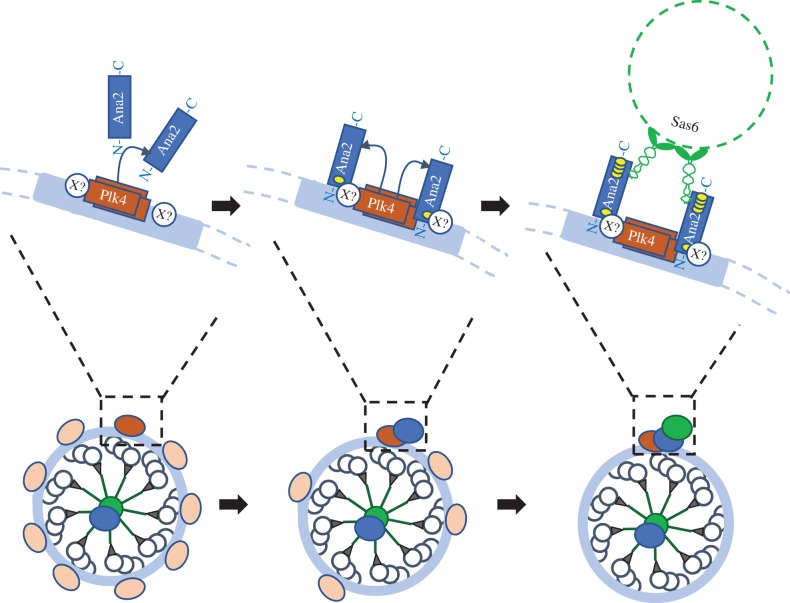
Sequential steps in the loading of Ana2 and Sas6 onto the site of the procentriole. Depiction of the spatial organization of a mother centriole (lower part) and the molecular interactions (upper part) of Plk4 (brown), Ana2 (blue) and Sas6 (green) during progression through anaphase (left), telophase (central), and late telophase-cytokinesis (right) in *Drosophila* cells. At the metaphase–anaphase transition, cells have a ring of Plk4 around the periphery of the centriole (Zone III) resembling a string of beads; Ana2 and Sas6 are present within the centriole core (Zone I). Plk4 will first phosphorylate Ana2 on S38 (single yellow site). In early telophase, Plk4 is lost from many, but not all, peripheral sites as a result of its proteolysis and Ana2 has begun to associate with one of the Plk4 sites, not necessarily the one with the highest levels of Plk4, but likely the one with the highest level of kinase activity (represented by the differential shading of Plk4). The Ana2 recruited to this site has been phosphorylated upon serine 38 in the ANST motif and associates either with Plk4 or another protein (X?, see Discussion). In late telophase-cytokinesis, Plk4 is lost from all but the procentriole site. Ana2 has been phosphorylated by Plk4 at four residues in the STAN motif (four yellow sites) and so can now bind to and recruit Sas6 dimers, which assemble into ninefold symmetrical rings.

Our study shows that Plk4 phosphorylates *Drosophila* Ana2 at a single serine residue, S38, in what we term the ANST motif in its N-terminal part to enable Ana2 to be recruited to the site of procentriole formation. This occurs ahead of the known requirement of Plk4 to phosphorylate Ana2 upon four residues in the conserved STAN motif in its C-terminal part in order to bind and recruit Sas6 to the pre-procentriole site [26]. Phosphorylation of the STAN motif is also required for Ana2's human counterpart, STIL, to bind and recruit Sas6 [27,28], where it also increases the efficiency of STIL's centriole targeting [28,35]. However, in neither human nor *Drosophila* cells is the phosphorylation of the STAN motif absolutely essential for STIL/Ana2 recruitment [26,27]. These findings led Holland and colleagues to suggest that Plk4 had a two-step function in initiating centriole duplication [28] although whether STIL had itself to be phosphorylated by Plk4 for its recruitment was unclear. Here we show that in *Drosophila* cells, the initiation of procentriole formation is, indeed, a two-step process requiring Plk4 to phosphorylate Ana2 at distinct sites (figure 7). Although the sites in the ANST and STAN motifs appear key for centriolar recruitment of Ana2 and binding to Sas6, respectively, it is noteworthy that Ana2 has numerous other Plk4 target sites. The functions, if any, of these additional sites are not yet clear but, at present, we cannot exclude the possibility of their importance to substantiate Ana2 recruitment or Sas6 binding, or to facilitate interactions with other centriole components.

Our findings have implications for the mechanisms that regulate the activation of Plk4 and that ultimately restrict it to a single site in normal cell cycle progression. The activation of Plk4 has consequences for its stability as well as for its ability to mediate its cellular functions including centriole duplication. This is because Plk4's destruction is brought about through the auto-phosphorylation of a degron rendering the kinase susceptible to SCF-mediated ubiquitylation and subsequent degradation by the proteasome [18–23]. But how is this balance between activation and destruction regulated on the centriole? Increased activity of the kinase has been proposed to occur as a consequence of its trans-autoactivation in response to its localized accumulation [36]. It has also been hypothesized that STIL might itself be an activator of Plk4 [28,35]. Indeed, overexpression of STIL was shown to trigger auto-phosphorylation of the activating T170 residue in Plk4's T-loop [28]. Our findings indicate that Plk4 is first recruited to multiple sites around mother and daughter centrioles before being restricted to a single procentriolar site. Although we demonstrate that recruitment to these multiple sites does not require the kinase to be catalytically active, its activity is required to trigger its autophosphorylation and SCF-mediated self-destruction from all but the single site to which Ana2 has been loaded. Our finding that Plk4 is still in the process of being eliminated from multiple sites in the periphery of the centriole, when Ana2 is already recruited to a single site would argue against a global requirement for STIL/Ana2 to trigger Plk4 activation [28,35] and more in favour of an auto-activation mechanism at sites from which it is subsequently eliminated.

Our results also suggest that a threshold level of Plk4 activity might be required to permit Ana2 loading. How this threshold becomes exceeded only at the single Ana2 loading site remains to be determined. Single-site loading of Ana2 could be related to the protection of Plk4 from destruction at the pre-procentriole site. It is possible that Plk4's binding to STIL/Ana2 might protect it from the SCF. This notion finds support from the finding that overexpression of wild-type STIL or a variant lacking the STAN motif can stabilize Plk4 in a ring around the centriole [27,35]. There is a growing consensus that the binding of STIL to Plk4 requires an interaction between a short coiled-coil motif in STIL and the L1 linker region and Polo-box 3 of Plk4 [27,35]. Indeed, a regulated interaction between Plk4 and STIL would seem to be important in regulating the timing of centriole formation by the finding that such an interaction is blocked until the metaphase-to-anaphase transition by Cdk1-mediated phosphorylation of STIL [37]. However, this exact same binding interface between Ana2 and Plk4 is reported to be absent in flies [38].

If Ana2 is recruited solely through binding to Plk4, then there must be a mechanism that restricts this interaction only to a single site. This could be achieved through a conformational change in one or both partners that could both reinforce and restrict the interaction. In this light, we note that Plk4 appears normally to exist in an autoinhibited state that is relieved through some property of Polo-box 3 suggested to reflect a conformational change resulting from binding a partner protein [39]. The shift in electrophoretic mobility we now show to be associated with phosphorylation of Ana2 on a site essential for its recruitment is likely to be important to lock the molecule into a conformation that is required for its recruitment. It remains a future challenge to determine whether phosphorylation of Ana2 on S38 and the associated change in its conformation enable Ana2 to associate with Plk4 or, as this has been questioned in *Drosophila*, whether it interacts with a different centriole component to allow it to dock and initiate procentriole formation. The conservation of the ANST motif is intriguing as it suggests the possibility, which will be of future interest to test, that Ana2 loading onto the procentriole site might be mediated by a conserved mechanism.

## Supplementary Material

Plk4 phosphorylation induces a band-shift in Ana2

## Supplementary Material

Serine-38 of Ana2 is phosphorylated in vitro by Plk4 as identified by mass-spectrometry.

## Supplementary Material

GST-Ana2-S38A can bind Sas6 after Plk4 phosphorylation

## Supplementary Material

Expression levels in Ana2-Myc cell lines

## Supplementary Material

List of site-directed mutagenesis primers

## Data accessibility

The datasets supporting this article have been uploaded as part of the electronic supplementary material.
