# Supplementary figures and images for: Two-step phosphorylation of Ana2 by Plk4 is required for the sequential loading of Ana2 and Sas6 to initiate procentriole formation
Source: Open Biol. 2017 Dec 20;7(12):170247. doi: 10.1098/rsob.170247 (PMC5746551; doi:10.1098/rsob.170247)

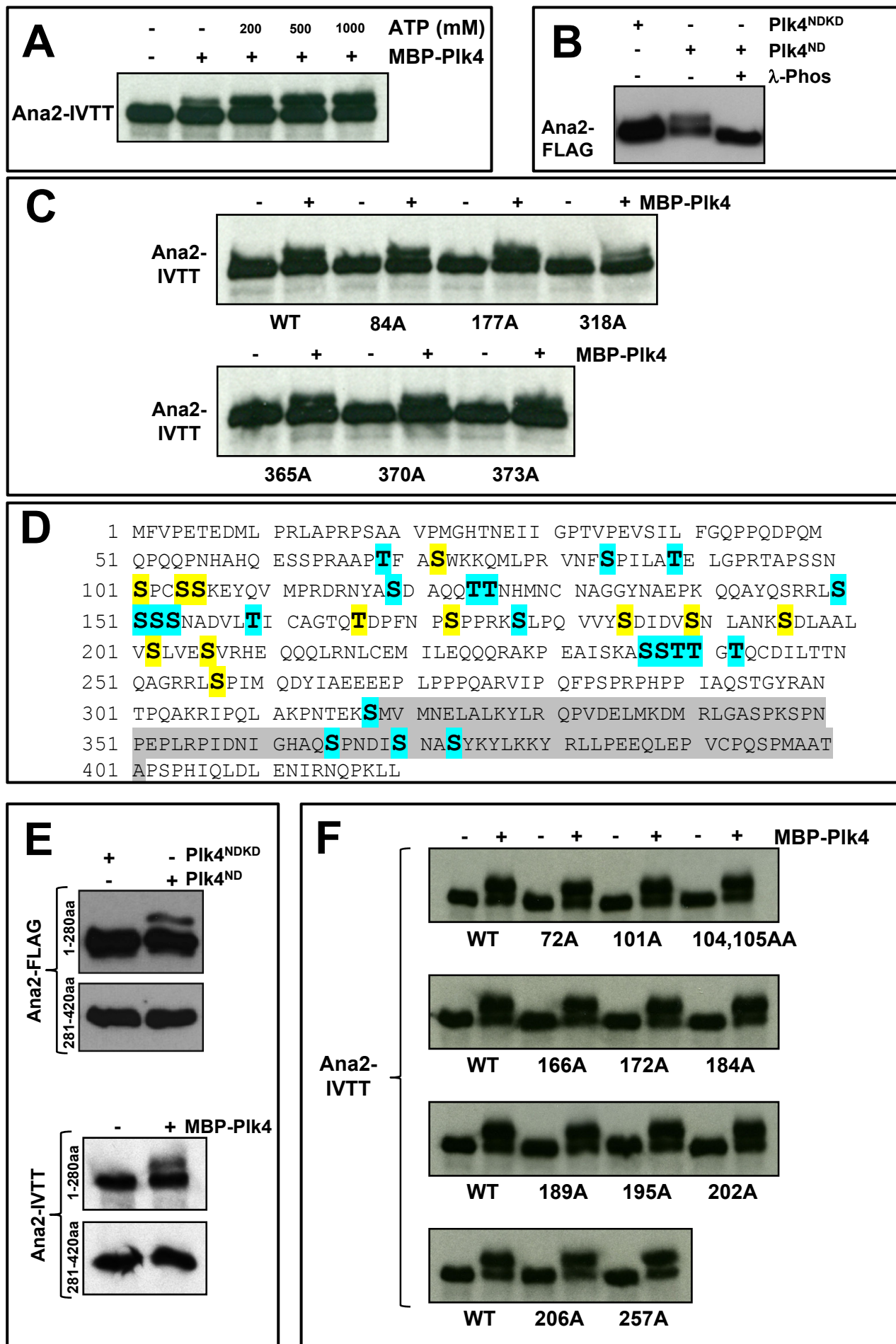

Suppl. Fig. S1

Supplement: Plk4 phosphorylation induces a band-shift in Ana2 [file rsob170247supp1.pdf]

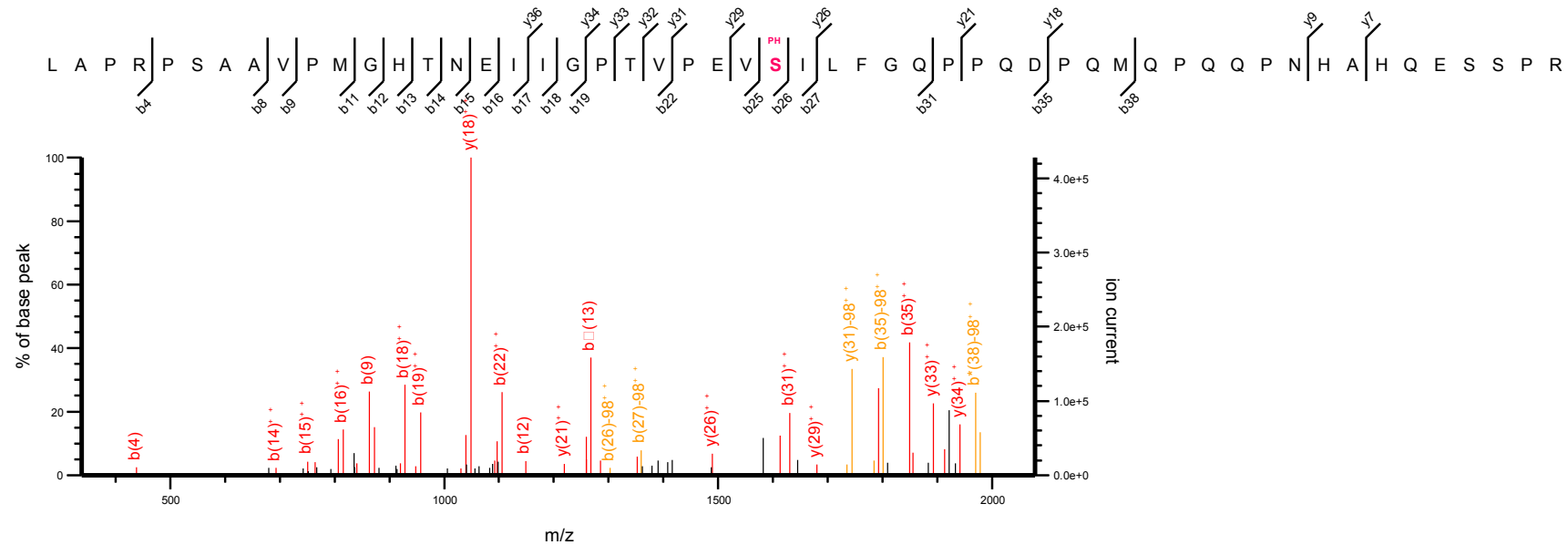

Suppl. Fig. S2

Supplement: Serine-38 of Ana2 is phosphorylated in vitro by Plk4 as identified by mass-spectrometry. [file rsob170247supp2.pdf]

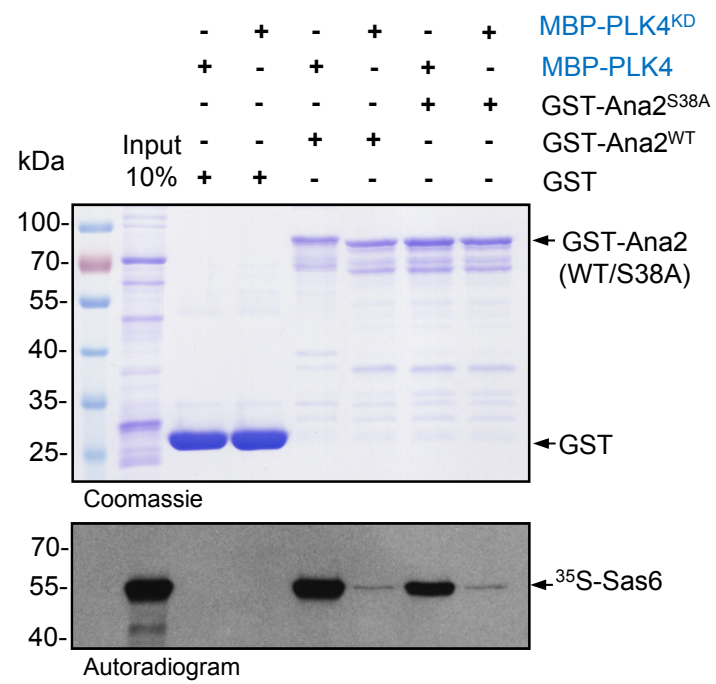

Suppl. Fig. S3

Supplement: GST-Ana2-S38A can bind Sas6 after Plk4 phosphorylation [file rsob170247supp3.pdf]

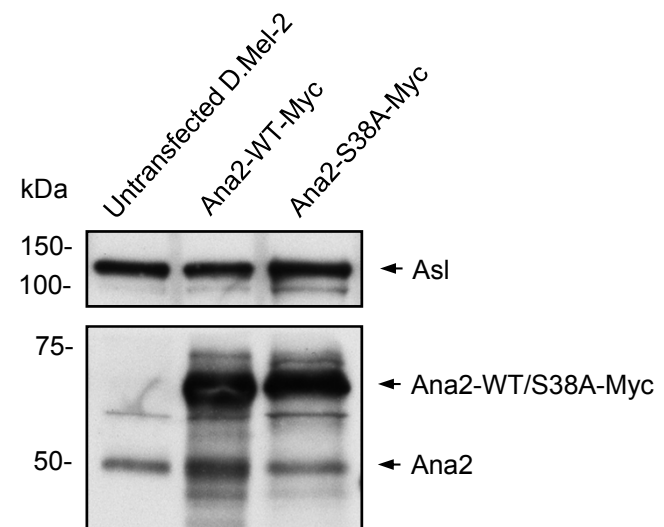

Suppl. Fig. S4

Supplement: Expression levels in Ana2-Myc cell lines [file rsob170247supp4.pdf]
