# Supplementary material for: Two-step phosphorylation of Ana2 by Plk4 is required for the sequential loading of Ana2 and Sas6 to initiate procentriole formation
Source: Open Biol. 2017 Dec 20;7(12):170247. doi: 10.1098/rsob.170247 (PMC5746551; doi:10.1098/rsob.170247)
Supplement: List of site-directed mutagenesis primers [file rsob170247supp5.pdf]

Ana2-T6A-F: 5’- CGCTCCAAATGTTTGTTCCCGAAGCAGAGGATATGCTGCCCAGACTAGC -3’
Ana2-T6A -R: 5’- GCTAGTCTGGGCAGCATATCCTCTGCTTCGGGAACAAACATTTGGAGCG -3’
Ana2-S18A -F: 5’- CCCAGACTAGCGCCCAGGCCGGCAGCAGCAGTGCCCATGGGCCAC -3’
Ana2-S18A -R: 5’- GTGGCCCATGGGCACTGCTGCTGCCGGCCTGGGCGCTAGTCTGGG -3’
Ana2-T26A -F: 5’- GCAGCAGTGCCCATGGGCCACGCAAATGAGATTATAGGGCCC -3’
Ana2-T26A -R: 5’- GGGCCCTATAATCTCATTTGCGTGGCCCATGGGCACTGCTGC -3’
Ana2-T33A -F: 5’- ACACCAATGAGATTATAGGGCCCGCAGTGCCGGAGGTATCCATATTGTT -3’
Ana2-T33A -R: 5’- AACAATATGGATACCTCCGGCACTGCGGGCCCTATAATCTCATTGGTGT -3’
Ana2-S38A-F: 5’- TAGGGCCCACCGTGCCGGAGGTAGCAATATTGTTTGGCCAGCCGCCGCA -3’
Ana2-S38A -R: 5’- TGCGGCGGCTGGCCAAACAATATTGCTACCTCCGGCACGGTGGGCCCTA -3’
Ana2-SS62,63AA -F: 5’- CCAAATCATGCACATCAGGAGGCAGCACCTCGTGCTGCACCCACCTTC -3’
Ana2-SS62,63AA -R: 5’- GAAGGTGGGTGCAGCACGAGGTGCTGCCTCCTGATGTGCATGATTTGG -3’
Ana2-T69A -F: 5’- GGAGTCATCCCCTCGTGCTGCACCCGCATTCGCGTCCTGGAAGAAGCAAATGC -3’
Ana2-T69A -R: 5’- GCATTTGCTTCTTCCAGGACGCGAATGCGGGTGCAGCACGAGGGGATGACTCC -3’
Ana2-S72A -F: 5’- CGTGCTGCACCCACCTTCGCGGCATGGAAGAAGCAAATGCTGCCC -3’
Ana2- S72A -R: 5’- GGGCAGCATTTGCTTCTTCCATGCCGCGAAGGTGGGTGCAGCACG -3’
Ana2-S84A-F: 5’- GCAAATGCTGCCCAGGGTCAACTTTGCACCCATTTTGGCCACAGAACTGGGTC -3’
Ana2-S84A -R: 5’- GACCCAGTTCTGTGGCCAAAATGGGTGCAAAGTTGACCCTGGGCAGCATTTGC -3’
Ana2-T89A -F: 5’- GGTCAACTTTTCGCCCATTTTGGCCGCAGAACTGGGTCCTCGCACCGCTCCCA -3’
Ana2-T89A -R: 5’- TGGGAGCGGTGCGAGGACCCAGTTCTGCGGCCAAAATGGGCGAAAAGTTGACC -3’
Ana2-SS98,99AA -F: 5’- CTGGGTCCTCGCACCGCTCCCGCAGCAAATAGTCCGTGCTCCTCGAAG -3’
Ana2-SS98,99AA -R: 5’- CTTCGAGGAGCACGGACTATTTGCTGCGGGAGCGGTGCGAGGACCCAG -3’
Ana2-S101A -F: 5’- CGCACCGCTCCCAGCAGCAATGCACCGTGCTCCTCGAAGGAATAC -3’
Ana2-S101A -R: 5’- GTATTCCTTCGAGGAGCACGGTGCATTGCTGCTGGGAGCGGTGCG -3’
Ana2-SS104,105AA-F: 5’- CCCAGCAGCAATAGTCCGTGCGCAGCAAAGGAATACCAAGTAATGCCC -3’
Ana2-SS104,105AA -R: 5’- GGGCATTACTTGGTATTCCTTTGCTGCGCACGGACTATTGCTGCTGGG -3’
Ana2-119,124,125-3xA -F: 5’- GTAATGCCCCGGGATCGCAACTATGCAGCAGATGCGCAGCAGGCAGC
AAATCATATGAACTGCAATGCTGGTGGC -3’
Ana2-119,124,125-3xA -R: 5’- GCCACCAGCATTGCAGTTCATATGATTTGCTGCCTGCTGCGCATCTGC
TGCATAGTTGCGATCCCGGGGCATTAC -3’
Ana2-150-153,159-5xA -F: 5’- CAACAGGCATATCAATCTAGGCGCTTGGCAGCAGCAGCAAACGCAGAT
GTCCTCGCAATCTGTGCTGGCACACAGACCGATCCC -3’
Ana2-150-153,159-5xA -R: 5’- GGGATCGGTCTGTGTGCCAGCACAGATTGCGAGGACATCTGCGTTTG
CTGCTGCTGCCAAGCGCCTAGATTGATATGCCTGTTG -3’
Ana2-T166A -F: 5’- ACCATCTGTGCTGGCACACAGGCAGATCCCTTCAACCCATCTCCT -3’
Ana2-T166A -R: 5’- AGGAGATGGGTTGAAGGGATCTGCCTGTGTGCCAGCACAGATGGT -3’
Ana2-T172A-F: 5’- CAGACCGATCCCTTCAACCCAGCACCTCCGCGAAAATCACTGCCC -3’
Ana2-T172A -R: 5’- GGGCAGTGATTTTCGCGGAGGTGCTGGGTTGAAGGGATCGGTCTG -3’
Ana2-S177A -F: 5’- CTTCAACCCATCTCCTCCGCGAAAAGCACTGCCCCAGGTTGTCTATTCCGACA -3’
Ana2-S177A -R: 5’- TGTCGGAATAGACAACCTGGGGCAGTGCTTTTCGCGGAGGAGATGGGTTGAAG -3’
Ana2-S184A -F: 5’- AAAATCACTGCCCCAGGTTGTCTATGCAGACATCGACGTCAGCAACTTGGCCA -3’
Ana2-S184A -R: 5’- TGGCCAAGTTGCTGACGTCGATGTCTGCATAGACAACCTGGGGCAGTGATTTT -3’
Ana2-S189A -F: 5’- GGTTGTCTATTCCGACATCGACGTCGCAAACTTGGCCAATAAGAGTGATCTGG -3’
Ana2-S189A -R: 5’- CCAGATCACTCTTATTGGCCAAGTTTGCGACGTCGATGTCGGAATAGACAACC -3’
Ana2-S195A-F: 5’- GTCAGCAACTTGGCCAATAAGGCAGATCTGGCGGCTCTGGTATCC -3’
Ana2-S195A -R: 5’- GGATACCAGAGCCGCCAGATCTGCCTTATTGGCCAAGTTGCTGAC -3’
Ana2-S202A -F: 5’- AGTGATCTGGCGGCTCTGGTAGCACTTGTGGAGTCCGTGCGCCAT -3’
Ana2-S202A -R: 5’- ATGGCGCACGGACTCCACAAGTGCTACCAGAGCCGCCAGATCACT -3’
Ana2-S206A -F: 5’- GCTCTGGTATCCCTTGTGGAGGCAGTGCGCCATGAGCAGCAACAG -3’
Ana2-S206A -R: 5’- CTGTTGCTGCTCATGGCGCACTGCCTCCACAAGGGATACCAGAGC -3’
Ana2-237-240,242-5xA -F: 5’- GCGAAACCCGAAGCCATTTCAAAAGCCGCAGCAGCAGCAGGCGCACAG
TGCGATATTCTGACCACCAATCAA -3’
Ana2-237-240,242-5xA -R: 5’- TTGATTGGTGGTCAGAATATCGCACTGTGCGCCTGCTGCTGCTGCGGCT
TTTGAAATGGCTTCGGGTTTCGC -3’
Ana2-S257A -F: 5’- AATCAAGCAGGTAGACGCTTGGCACCCATTATGCAGGATTACATA -3’
Ana2-S257A -R: 5’- TATGTAATCCTGCATAATGGGTGCCAAGCGTCTACCTGCTTGATT -3’

Suppl. Table 1
